# The Immunotoxicity of Chronic Exposure to High Levels of Lead: An Ex Vivo Investigation

**DOI:** 10.3390/toxics8030056

**Published:** 2020-08-13

**Authors:** Kawinsaya Pukanha, Supabhorn Yimthiang, Wiyada Kwanhian

**Affiliations:** 1 Department of Medical Technology, School of Allied Health Sciences, Walailak University, Nakhon Si Thammarat 80161, Thailand; p.kawinsaya@gmail.com; 2 School of Public Health, Walailak University, Nakhon Si Thammarat 80160, Thailand; ksupapor@mail.wu.ac.th

**Keywords:** blood lead, cellular immunity, phagocytosis, humoral munity, immunosuppression

## Abstract

Lead (Pb) is a toxic metal known for its wide-ranging adverse health effects. However, a compound of Pb is still used in the caulking process to repair wooden fishing boats. The present study aimed to measure Pb exposure and its immunologic effects in boatyard workers in Nakhon Si Thammarat province, Thailand, in comparison with an age-matched control group of farmers. The age, body mass index, and smoking history in workers (*n* = 14) and controls (*n* = 16) did not differ. The median blood Pb concentration was 8.7-fold higher in workers than controls (37.1 versus 4.3 µg/dL, *p* < 0.001). Workers had 8.4% lower phagocytic active cells than controls (89.9% versus 98.1%, *p* = 0.019). In response to a mitogen stimulation, the peripheral blood mononuclear cells (PBMCs) from workers produced 2-fold higher ratios of interleukin-4 (IL-4) to interferon-γ than the PBMCs from controls (*p* = 0.026). Furthermore, Pb-exposed workers had 33.9% lower cytotoxic T (Tc) cells than controls (24.3% versus 36.8%, *p* = 0.004). In stark contrast, the percentage of regulatory T (Treg) cells in workers was 2.7-fold higher than controls (6.1% versus 2.3%, *p* < 0.001). In all subjects, blood Pb showed positive correlations with the percentages of Treg cells (*r* = 0.843, *p* < 0.001) and IL-4 (*r* = 0.473, *p* = 0.041) while showing an inverse correlation with the percentages of Tc cells (*r* = −0.563, *p* = 0.015). These findings indicate that chronic high Pb exposure may cause a shift towards humoral immune response, together with a suppression of cellular immunity, thereby suggesting an elevation in cancer risk in Pb-exposed workers.

## 1. Introduction

Lead (Pb) is a heavy metal with versatile properties, including malleability, ductility, poor conductivity, softness, and corrosion resistance, and it has thus been used for several thousands of years, causing widespread distribution in the environment worldwide [1,2]. Due to its non-biodegradability, Pb accumulates in the environment and hazards increase over time, evident from numerous reports of Pb contamination in the environment by industrial, mining, and agricultural activities [1,2,3]. Pb has no known biological role, but it can accumulate in the body, causing toxicity in many tissues and organs, immune system included [1,2,3,4,5,6,7,8,9,10,11,12]. It is of concern that there is increasing evidence for the negative effects of chronic exposure to high-level Pb on cancer risk and mortality that has recently emerged from prospective cohort studies of Pb-exposed workers [13,14]. 

The immune system provides important defense mechanisms that reduce the potential adverse effects of exposure to harmful biological agents, mutant cells, and certain chemicals [15]. Studies in humans and experimental animals have demonstrated the adverse effects of Pb exposure on the body’s immune system [16,17,18,19,20,21]. In one study, Pb-poisoned children were found to have lower numbers of CD4+CD8+ helper T cells (Th), while their CD3+CD8+ cytotoxic T cell (Tc) levels were higher than those of a control group [16]. Workers in a factory manufacturing lead stearate were found to have a decreased number of CD45RO+ memory T cells [17]. A decrease in lymphocyte proliferation was noted in another study of occupationally exposed workers [18]. A reduction in humoral immunity by 53.6% was observed in mice receiving Pb in drinking water [19]. In other studies, altered subpopulations of circulating CD4+ T cells (T cells) and CD8+ T cells (T cytotoxic cells) were noted, together with evidence for Th1 up-regulation occurring simultaneously with Th2 down-regulation [20,21].

Nakhon Si Thammarat Province, located in the southern region of Thailand, is not an industrial estate area. However, there are many shipyards in operation, especially in coastal areas such as the districts of Pak Phanang, Mueng, Thasala, and Si-Chon. Many occupations are associated with the shipyards, including boat caulkers and fishing net workers, using lead bars [22]. In a previous report published in 2007, 48% to 67% of caulkers, painters, and mechanics working in the shipyards of Pak Phanang and Thasala districts were found to have blood Pb concentrations >40 µg/dL [23]. The present study was undertaken to assess Pb exposure experienced by current boatyard workers by measuring their blood Pb concentrations. In addition, it examined the adverse effects of such exposure, with a focus on the function of immune cells, which included phagocytic activity, proliferation, and cytokine production, and T cell subpopulation profiles. 

## 2. Materials and Methods

### 2.1. Study Subjects

The Committee on Human Rights Related to Research Involving Human Subjects at Walailak University approved all experimental protocols in the present study (approval no. 14/057, 1 August 2014). A total of fourteen Pb-exposed workers (boat caulkers and fishing net workers included) from Pak Phanang district, Nakhon Si Thammarat Province, Thailand, were enrolled in this study. Additional age-matched farmers (*n* = 16) were enrolled as controls. The main criterion for inclusion in the worker group was blood Pb concentration ≥25 µg/dL. For controls, blood Pb levels were <25 µg/dL. The blood Pb level of 25 µg/dL was an exposure limit for Pb-exposed workers, based on the Occupational Safety and Health Administration (OSHA) [24]. 

### 2.2. Sampling and Blood Analysis

From each subject, a 50-mL blood sample was drawn from the median cubital vein in the morning, before working and without fasting. The blood sample of each subject was divided into three aliquots of 5, 10, and 35 mL. The 5-mL aliquots of whole blood, in BD Vacutainer® EDTA tube (Becton, Dickinson and Company, Franklin Lakes, NJ, USA) were assayed for Pb concentrations, using the graphite furnace atomic absorption spectrometer, AAnalyst^TM^ 600 (PerkinElmer, Wellesley, MA, USA), available at Toxicology Laboratory, Department of Pathology, Faculty of Medicine, Ramathibodi Hospital, Mahidol University, Bangkok, Thailand. Whole Blood Metals Control Lymphochek^TM^ Levels 1, 2 and 3 were used for quality assurance and control (Bio-Rad, Hercules, CA, USA). The coefficients of variation of blood Pb concentrations were within 10%. None of study subjects had blood Pb concentrations below the detection limit of 1.0 µg/dL [25]. The 10-mL aliquots of blood samples contained heparin as an anticoagulant were assayed for T cell subpopulations and phagocytic activity. The 35-mL aliquots of blood samples contained heparin as an anticoagulant were subjected to preparation of mononuclear cells, as detailed in Section 2.3.

### 2.3. Isolation of Peripheral Blood Mononuclear Cells (PBMCs)

PBMCs were isolated by the Ficoll density gradient centrifugation method [26] from 35-mL aliquots of blood samples, collected in BD Vacutainer® Lithium Heparin tubes (Becton, Dickinson and Company, Franklin Lakes, NJ, USA). Cells were counted and cell viability was determined by trypan blue staining. The PBMCs 2 × 10^6^ cells/mL were cultured in RPMI 1640 Gibco^TM^ culture medium (Thermo Fisher, Waltham, MA, USA) supplemented with 15% fetal bovine serum (Merck, Darmstadt, HE, Germany) and 1% penicillin-streptomycin Gibco™ (Thermo Fisher Scientific, MA, USA).

### 2.4. Phagocytic Activity Assay

Phagocytosis activity was determined using the IgG-FITC Phagocytosis assay kit (Cayman Chemical, Ann Arbor, MI, USA). The instructions in the manufacturing manual were slightly modified. One milliliter of buffy coat from heparinized blood was mixed with 9 mL FACS^TM^ lysing solution (Becton Dickinson and Company, Franklin Lakes, NJ, USA), and the samples were incubated for 15 min at room temperature. The cells were centrifuged for 5 min at 400× *g* at room temperature. The supernatant was carefully decanted, and cells were adjusted with PBS to a concentration of 1 × 10^6^ cells/mL. Cells were transferred into polypropylene FACS^TM^ tubes (Becton Dickinson and Company, Franklin Lakes, NJ, USA) and mixed with the latex beads rabbit IgG-FITC solution. Subsequently, cells were incubated in the dark in an incubator with 5% CO_2_, 37 °C for 1 h. The percentages of active phagocytic cells were determined by BD FACSCalibur^TM^ flow cytometry (Becton Dickinson and Company, Franklin Lakes, NJ, USA).

### 2.5. Cytokine Assay

To stimulate cytokine production, separated PBMCs were mixed with phytohemagglutinin (PHA) at a concentration of 5µg/mL (Merck, Darmstadt, HE, Germany) before they were incubated at 5% CO_2_, 37 °C for 48 h [21]. Supernatants were harvested from each well and stored at −80 °C until they were measured for levels of cytokines, namely interleukin-4 (IL-4) and interferon-γ (IFN-γ), by ELISA human cytokine development kits (PeproTech, Rocky Hill, NJ, USA). Each sample was analyzed in triplicate, and controls and unstimulated blanks were analyzed simultaneously.

### 2.6. Proliferation Assay

A total of 2 × 10^6^ cells/mL of PBMCs in complete medium were grown in 96-well plates and stimulated with 5 µg/mL PHA [13] and incubated at 37 °C, 5% CO_2_ for 48 h. Following incubation, 3-(4-5-dimethylthaizolyl-2)-2,5-diphenyltetrazolium bromide (Merck, Darmstadt, HE, Germany) was added and the cells were incubated at 37 °C for a further 4-h period. Then, 100 µL of dimethyl sulfoxide (DMSO) was added and samples were mixed thoroughly by repeated pipetting and incubated at room temperature in the dark for 2 h. The absorbance wavelength of 570 nm and the reference wavelength of 630 nm were measured by a Thermo Scientific^TM^ Multiskan^TM^ GO microplate reader (Thermo Fisher Scientific, Waltham, MA, USA). Each sample was analyzed in triplicate, and controls and unstimulated blanks were analyzed simultaneously.

### 2.7. Determination of T Cell Subpopulations by the Flow Cytometry

Cells were stained before they were incubated with FACS lysing solution, containing paraformaldehyde for fixing cells (https://www.bdbiosciences.com/ds/is/tds/23-1358.pdf). To control for staining variability, negative isotype control was used for every sample tested. The negative isotype control ensured that the observed staining was due to specific antibody binding to the target rather than an artefact or background. The utility of Fc receptor blocking reagents was unnecessary when the negative isotype was used. For CD4 and CD8, at least 10,000 event cells were collected as the concentrations of Th and Tc cells were 800 and 500 cells/µL, respectively. For Treg cells, at least 20,000 events were collected as there was 1–2% of CD4 T cells.

#### 2.7.1. Helper T Lymphocytes and Cytotoxic T Lymphocytes

To a 5-mL Falcon^TM^ polystyrene tube (Becton Dickinson and Company, Franklin Lakes, NJ, USA), fifty microliters of EDTA blood was added per 10 µL of BD Tritest CD4/CD8/CD3 (Becton Dickinson and Company, Franklin Lakes, NJ, USA). The method followed the manufacturer’s manual. The negative control tube was reacted with the BD FastImmune^TM^ γ1 PE/CD45 PerCP control (Becton Dickinson and Company, Franklin Lakes, NJ, USA). Samples were analyzed by BD FACSCalibur^TM^ flow cytometry (Becton Dickinson and Company, Franklin Lakes, NJ, USA). Cytotoxic T (Tc) cells are CD3+CD8+, while helper T (Th) cells are CD3+CD4+. The data were analyzed by BD CellQuest software version 5.0, 2002 (Becton Dickinson and Company, Franklin Lakes, NJ, USA).

#### 2.7.2. Regulatory T Lymphocytes

To a 5-mL Falcon^TM^ round-bottom polystyrene tube (Becton Dickinson and Company, Franklin Lakes, NJ, USA), fifty microliters of buffy coat of EDTA blood was added per 10 µL of isotype control (PE-Cy^TM^ 7 Mouse IgG1κ isotype control (Becton Dickinson and Company, Franklin Lakes, NJ, USA) and cocktail of human regulatory T cells (CD4/CD25/CD127) (Becton Dickinson and Company, Franklin Lakes, NJ, USA) antibody in each sample. The regulatory T (Treg) cells (CD4+CD25^bright^CD127^dim^) were analyzed by flow cytometry. Events collected using a FACSCalibur^TM^ (Becton Dickinson and Company, Franklin Lakes, NJ, USA) were analyzed using CellQuest version 5.0, 2002 (Becton Dickinson and Company, Franklin Lakes, NJ, USA).

### 2.8. Statistical Analysis

GraphPad Prism software version 8.01, 2018 (GraphPad Software, San Diego, CA, USA) was used to analyze data. The Mann–Whitney U test was used to determine differences in age, body mass index (BMI), and blood Pb concentration in controls versus workers. Difference in smoking history was determined by odds ratio and chi-square test. Spearman’s rank correlation was used to identify the correlations between blood Pb concentration and seven measured immunologic parameters, including proliferation index, percentages of phagocytic cells, levels of cytokines, and percentages of T cell subpopulations. The result was considered statistically significant when *p*-values were less than 0.05 in a two-sided test.

## 3. Results

### 3.1. Characteristics of Study Workers and Controls

Table 1 provides the characteristics of the control group and worker group. Participants in the control group were men, while the worker group had nine men and five women. Half of the study workers were exposed to Pb for more than 10 years. Age, BMI, hemoglobin, hematocrit, and smoking history of both groups did not differ. Thus, demographic characteristics of the two groups were apparently homologous. The median blood Pb concentration in the worker group was 37.07 μg/dL. This blood Pb concentration was 8.7-fold higher than the controls (*p* < 0.001). It was also higher than the OSHA exposure limit for Pb-exposed workers of 25 μg/dL [24]. 

### 3.2. Profiling of Cytokine Production and Other Immunologic Parameters

Table 2 provides a profile of the immunologic parameters measured. These included the percentage of helper T (Th) lymphocytes (CD3+CD4+) together with cell proliferation index and cytokine production in response to stimulation by a mitogen. The median percentage of Th lymphocytes in workers was similar to that of controls (60.74% versus 56.31%, *p* = 0.395). In the cell proliferation and cytokine production assay, peripheral blood mononuclear cells (PBMCs) from study subjects were used, with PHA as a stimulant mitogen. The median PHA-stimulated cell proliferation index was 1.14 in workers and 1.50 in controls (*p* = 0.226). The median level of interferon-gamma (IFN-γ) generated in response to PHA stimulation was 12.14 pg/mL in workers and 20.07 pg/mL in controls (*p* = 0.103), while the median level of interleukin-4 (IL-4) was 175.58 pg/mL in workers and 155.65 pg/mL in controls (*p* = 0.060). Interestingly, the median ratio of IL-4/IFN-γ was approximately 2-fold higher in workers compared with controls (15.03 versus 7.01, *p* = 0.026). Hence, there was a shift towards humoral immune response in Pb-exposed workers, evident from an increase in IL-4 production concomitant with a decrease in IFN-γ production, which has a suppressive effect on cell-mediated immunity.

### 3.3. Effects of High Pb Exposure on Innate Immunity

**Figure 1:**
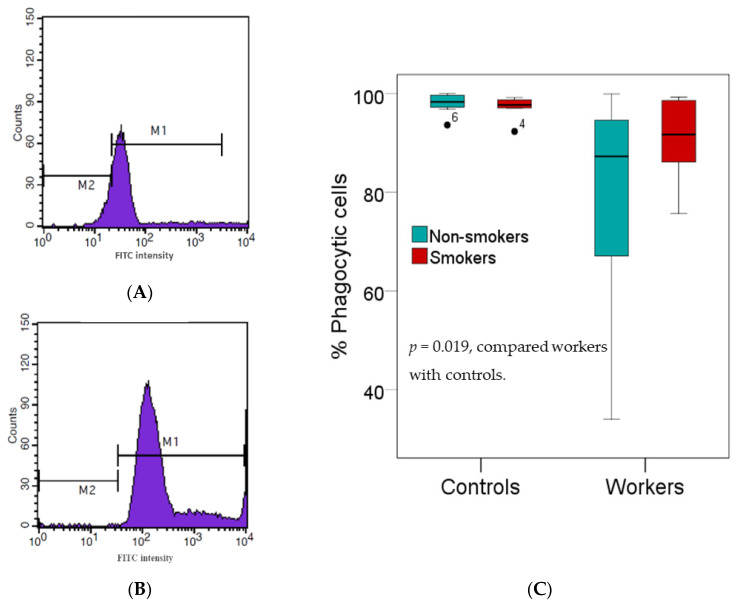
Effects of high Pb exposure on innate immunity. Histograms show the percentages of phagocytic cells in one Pb-exposed worker (**A**) and one control subject (**B**). Boxplots compare percentages of phagocytic cells in controls versus workers, non-smokers, and smokers included (**C**). Outliers were the data points below or above 1.5× interquartile range. The *p*-value was derived from the Mann–Whitney U test, in which outliers were included.

Figure 1 provides results of an investigation on innate immunity, where white blood cells separated from the buffy coats of fresh heparinized blood samples from subjects who were tested for their phagocytic activity. As the effect of smoking on the percentage of phagocytic cells was minuscule, data from non-smokers and smokers in the group of workers or controls were pooled. The median percentage of phagocytic active cells was 8.4% lower in workers than controls (89.92% vs. 98.11%, *p* = 0.019).

### 3.4. Effects of High Pb Exposure on the Populations of Cytoxic and Regulatory T Lymphocytes

**Figure 2:**
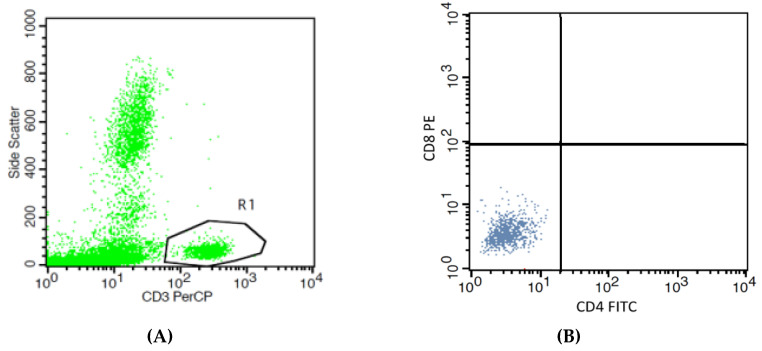

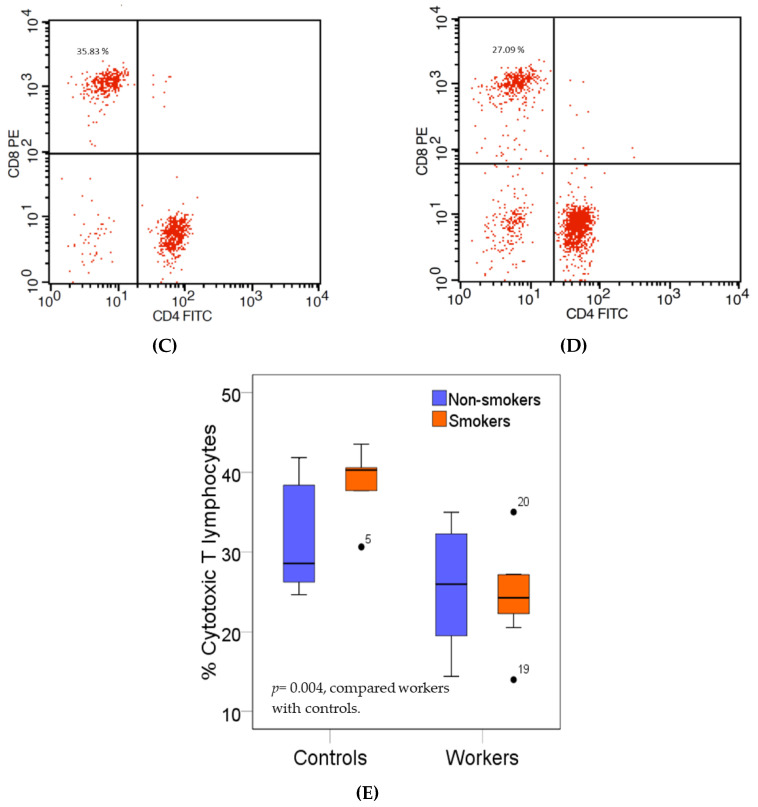
Effects of high Pb exposure on the percentage of cytotoxic T lymphocytes. The flow cytometry strategies (dot plots) show side scatter vs. CD3 (PerCP) (**A**), isotype control stained (**B**), the cytotoxic T (Tc) lymphocytes in one control subject (**C**), and one Pb-exposed worker (**D**). Boxplots compare percentages of Tc lymphocytes in controls versus workers, non-smokers, and smokers included (**E**). Outliers were the data points below or above 1.5× interquartile range. The *p*-value was derived from the Mann–Whitney U test, in which outliers were included.

Figure 2 provides boxplots that compare the percentages of cytotoxic T (Tc) lymphocytes (CD3+CD8+) in controls and workers, stratified by smoking status. As an effect of smoking on percentage of Tc lymphocyte was statistically insignificant, data from non-smokers and smokers in the group of workers or controls were pooled. The median percentage of Tc lymphocytes in the worker group was 33.9 % lower than the control group (24.30% versus 36.76%, *p* = 0.004).

**Figure 3:**
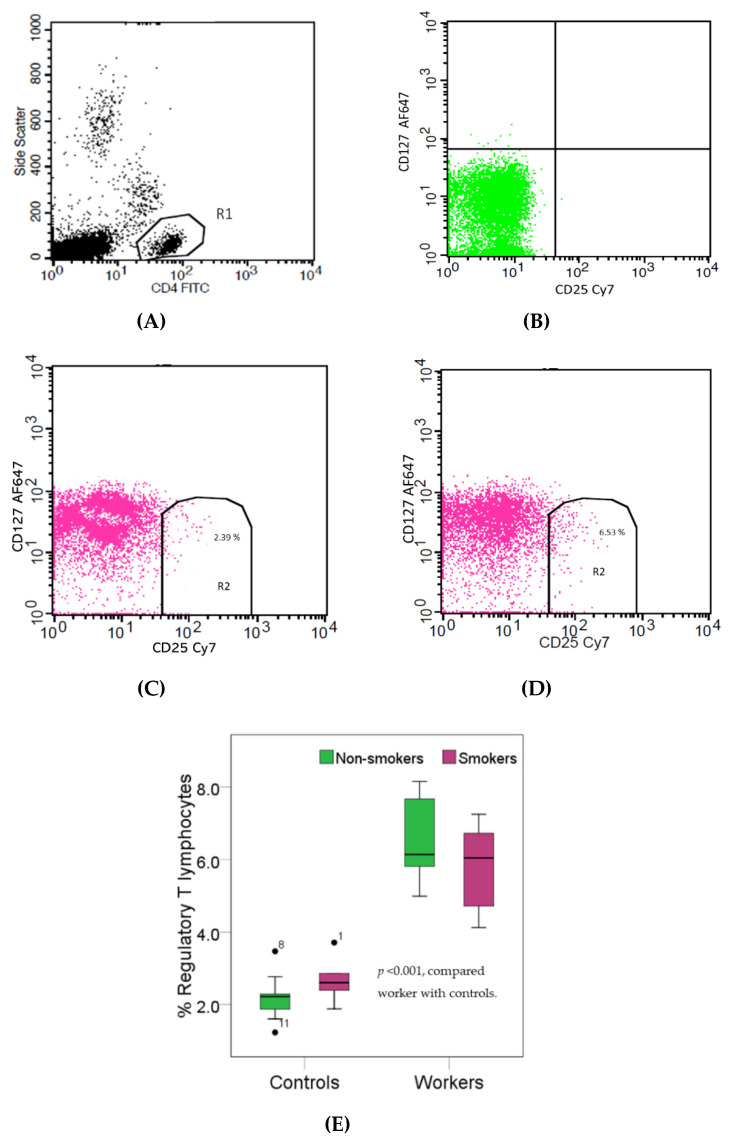
Effects of high Pb exposure on the percentage of regulatory T lymphocytes. The flow cytometry strategies (dot plots) show side scatter vs. CD4 (FITC) (**A**), isotype control stained (**B**), the regulatory T (Treg) lymphocytes in one control subject (**C**) and one Pb-exposed worker (**D**). Boxplots compare percentages of regulatory Treg lymphocytes in controls versus workers, non-smokers, and smokers included (**E**). Outliers were the data points below or above 1.5× interquartile range. The *p*-value was derived from the Mann–Whitney U test, in which outliers were included.

Figure 3 provides boxplots that compare the percentages of regulatory T (Treg) lymphocytes (CD4+CD25^bright^CD127^dim^) in controls and workers, stratified by smoking status. As the effect of smoking on the percentage of Treg lymphocytes was statistically insignificant, data from non-smokers and smokers in the group of workers or controls were pooled. The median percentage of Treg lymphocytes in the worker group was 2.7-fold higher than the control group (6.10% versus 2.28%, *p* < 0.001). Pb-exposed workers appeared to have a decreased percentage of Tc cells concomitantly with an increased percentage of Treg cells, thereby suggesting suppression of cellular immunity.

### 3.5. Blood Pb in Relation to Cytokines amd Immunologic Parameters

Table 3 provides results of the Spearman’s rank correlation analysis of blood Pb concentration and seven immunologic parameters measured for an entire group (controls plus workers, *n* = 30). Blood Pb concentration did not correlate with phagocytic activity, proliferative response, IFN-γ, or % T helper cells (*p* ≥ 0.05). However, blood Pb concentrations showed significant positive correlations with the percentages of Treg cells (*r* = 0.843, *p* < 0.001) and IL-4 (*r* = 0.473, *p* = 0.041), while showing an inverse correlation with the percentages of Tc cells (*r* = −0.563, *p* = 0.015). Thus, Pb-exposed workers were found to have elevated IL-4 production levels together with an elevated percentage of Treg cells, while having a decreased percentage of Tc cells.

## 4. Discussion

In this study, we investigated Pb exposure levels in occupational settings together with adverse effects of such exposure levels on the function of the body’s immune system. The blood Pb concentrations in study workers showed a wide range, 25.11 to 58.47 μg/dL, while the blood Pb concentrations in age-matched farmers (a control group) were in a narrow range, 3.02 to 4.24 μg/dL. The most likely source of Pb in the control group is the diet, as Pb is present in virtually all foodstuffs [27,28]. The effects of Pb on lymphocyte proliferation, natural killer (NK) cell cytotoxicity, and IFN-γ production by PBMCs have been seen in occupationally exposed persons [29]. In parallel, some effects of Pb on the immune response have been seen in animal models, such as the innate immune system in zebra fish [30] and the humoral and cell-mediated immune responses in mice [31,32]. 

Compared with a former report [23], Pb exposure levels among boatyard workers in our study workers remained as high as previously observed. In addition, we observed a decrease in phagocytic activity of the neutrophils from boatyard workers. This finding agreed with a previous report [31]. The decreased phagocytic activity may be attributable to the action of IL-1, as it is known to be involved in the stimulation of neutrophils and the recruitment activated cells into the site of injury [33]. However, IL-1 was up-regulated after exposure to mixtures of Pb and arsenic [30].

IFN-γ, produced by CD8+ Th1 cells, represents a component of cell-mediated immunity known for its anti-viral and anti-parasitic propensities [34]. IFN-γ inhibits the proliferation of Th2 cells and acts in synergy with other cytokines, notably TNF-α, to impede the proliferation of normal and transformed cells [34]. In a recent review, in vivo, in vitro, and ex vivo studies have been used to confirm that IFN-γ production is inhibited by Pb [35]. It is of relevance that the levels of IFN-γ produced by PBMCs from workers showed a tendency to be lower than those from controls. In an early study [36], Pb acetate was found to be involved in immediate hypersensitivity reactions (degranulation of rat mast cells) and allergic hypersensitivity, which was mediated by Th2 [36]. Exposure to low-dose Pb led to a decrease in IFN-γ Th1 cytokine and proinflammatory cytokines TNF-α and IL-1 while inducing IL-4 and/or IL-10 to maintain a Th2 immune response [37]. It has been suggested that Pb may increase susceptibility to infection and the incidence of allergic hypersensitivity [35,37]. 

IL-4 is a Th2 cytokine secreted by activated Th2 and NKT cells. It is a potent inducer of naïve CD4+ T cells and directs their differentiation into Th2 effector cells. IL-4 is known as a marker of humoral immunity [38]. In the present study, PBMCs from workers showed a tendency to produce more IL-4 together with less IFN-γ than controls. Consequently, the IL-4/IFN-γ ratio produced by PBMCs from workers was 2-fold higher than the control group. This indicated toxicity of Pb on cytokine production, which is consistent with previous studies in the following ways. In BALB/c mice, levels of the *IL-4* gene were increased after exposure to heavy metals, Pb included [39]. Elevated IL-4 after Pb exposure may cause an induction of type 2 helper T (Th2) cells and M2 macrophages [40]. In effect, the increased production of IL-4 seen in boatyard workers might confer upon them increased susceptibility to infection, allergic hypersensitivity, and/or autoimmune diseases dominated by Th2. 

A significant increase in Treg observed among workers in the present study is similar to previous experimental studies [41,42]. Up-regulation of IL-2RB in lymphocytes after exposure to heavy metals was observed in addition to the higher expression of Treg differentiation [43,44]. It is suggested that Pb may activate TGF-β, one of the main regulatory cytokines [41], which in turn stimulates the differentiation of regulatory T cells, promoting FoxP3 expression [45]. The link between Treg cells and cytokine suppression was confirmed by a study targeting the PI3-AKT pathway that caused the inhibition Treg proliferation [46]. In the present study, a significant reduction in Tc lymphocytes (CD3+CD8+) was observed in boatyard workers. Likewise, a significant decrease in the percentage of CD4+ Th cells was noted in a study of Pb-exposed children [47].

## 5. Conclusions

Herein, we have demonstrated that boatyard workers continue to be exposed to toxic high levels of Pb, as reflected by their blood Pb levels, which are 8.7-fold higher than the farmer control group. The immunological effects associated with such toxic exposure levels among study workers are reduced phagocytic activity, altered cytokine profiles (an increase in IL-4 concomitant with a decrease in IFN-γ), and deranged subpopulations of Tc and Treg cells, causing suppression of cell-mediated immunity. These findings may explain the increased risk of death from cancer and increased incidence of lung cancer and brain cancer in workers with high exposure, seen in cohort studies of Pb-exposed workers [13,14]. Public measures are required in order to reduce workplace exposure, as is a further study with a larger sample size to substantiate these important observations. 

## Figures and Tables

**Table 1:** Demographic data of control and worker groups.

Descriptors	All Subjects	Controls	Workers	Odds Ratio	*p*-Value ^1^
Number	30	16	14		
Age, years	53.0 ± 9.7	51.5 ± 8.8	54.0 ± 11.3	-	0.595
Range, years	28–69	36–66	28–69		
BMI, kg/m^2^	22.9 ± 4.1	23.7 ± 4.7	24.0 ± 4.6	-	0.874
Range, kg/m^2^	16.9–32.5	19.1–32.5	16.9–30.3		
Hemoglobin, g/dL	13.9 ± 1.2	14.1 ± 1.1	13.4 ± 1.2	-	0.550
Range, g/dL	11.7–16.4	12.2–16.4	11.7–15.6		
Hematocrit, %	41.8 ± 3.2	42.4 ± 2.9	40.9 ± 3.0	-	0.698
Range, %	36.3–48	37.7–48	36.3–45.2		
Blood Pb concentration, µg/dL	16.17 ± 18.68	4.28 ± 1.12	37.07 ± 11.09	-	<0.001
Range, µg/dL	3.02–58.47	3.02–4.24	25.11–58.47		
Sex				-	-
Male	25	16	9		
Female	5	0	5		
Occupation				-	-
Caulker	10	0	10		
Fishing net worker	4	0	4		
Agriculturist	16	16	-		
Duration of Pb exposure				-	-
0 year	16	16	0		
1–5 years	3	0	3		
5–10 years	4	0	4		
>10 years	7	0	7		
Smoking history				1.158	0.282
Yes	14	6	8		
No	16	10	6		

Age, BMI, hemoglobin, hematocrit, and blood Pb concentration are presented as median ± standard deviation (SD).—means no data or not determined. ^1^ The *p*-values for age, BMI, and blood Pb were derived from the Mann–Whitney U test. The *p*-value for smoking history was derived from chi-square test. Significant different at *p*-value < 0.05.

**Table 2:** Profiling of immunologic parameters.

Parameters	Group	Median	SD	Range	95% CI	*p*-Value ^1^
Th (%)	Controls	56.3	10.1	41.3–78.4	51.9–68.4	0.395
	Workers	60.7	9.3	45.8–77.2	56.9–69.9	
Proliferation index	Controls	1.50	0.35	1.06–2.41	1.22–1.66	0.226
	Workers	1.14	0.26	1.09–1.66	1.09–1.66	
IFN-γ (pg/mL)	Controls	20.1	9.3	3.2–30.0	3.2–30.0	0.104
	Workers	12.1	4.6	3.6–20.6	10.0–15.5	
IL-4 (pg/mL)	Controls	155.6	40.0	103.4–252	112.1–163	0.060
	Workers	175.6	67.2	99.2–334	114.7–215	
IL-4/IFN-γ ratio	Controls	7.0	15.6	3.5–50.4	3.5–50.4	0.026
	Workers	15.0	11.8	6.1–48.1	10.3–31.3	

^1^ The *p*-value was derived from the Mann–Whitney U test, significant difference at *p*-value < 0.05.

**Table 3:** The Spearman’s rank correlation analysis of blood Pb concentrations and seven immunologic parameters.

Blood Pb Versus Immunologic Parameters	Phagocytic Activity	Proliferation Index	IFN-γ	IL-4	%Th	%Tc	%Treg
Spearman’s rho	−0.209	−0.329	−0.319	0.473	0.358	−0.563	0.843
*p*-value (two-tailed)	0.364	0.231	0.213	0.041	0.121	0.015	<0.001
Significant (α = 0.05)	No	No	No	Yes	No	Yes	Yes

## References

[1] Tchounwou P.B., Yedjou C.G., Patlolla A.K., Sutton D.J. (2012). Heavy metal toxicity and the environment. Exp. Suppl..

[2] de Souza I.D., de Andrade A.S., Dalmolin R.J.S. (2018). Lead-interacting proteins and their implication in lead poisoning. Crit. Rev. Toxicol..

[3] Caito S., Aschner M. (2017). Developmental neurotoxicity of lead. Adv. Neurobiol..

[4] Li X., Gao Y., Zhang M., Zhang Y., Zhou M., Peng L., He A., Zhang X., Yan X., Wang Y. (2020). In vitro lung and gastrointestinal bioaccessibility of potentially toxic metals in Pb-contaminated alkaline urban soil: The role of particle size fractions. Ecotoxicol. Environ. Saf..

[5] Liu J., McCauley L., Yan C., Shen X., Pinto-Martin J.A. (2012). Low blood lead levels and hemoglobin concentrations in preschool children in China. Toxicol. Environ. Chem..

[6] Hashem M.A., El-Sharkawy N.I. (2009). The effects of low electromagnetic field and lead acetate combination on some hemato-biochemical and immunotoxicological parameters in mice. Turk. J. Hematol..

[7] Valcke M., Ouellet N., Dubé M., Laouan Sidi E.A., LeBlanc A., Normandin L., Balion C., Ayotte P. (2019). Biomarkers of cadmium, lead and mercury exposure in relation with early biomarkers of renal dysfunction and diabetes: Results from a pilot study among aging Canadians. Toxicol. Lett..

[8] Kim Y.D., Eom S.Y., Yim D.H., Kim I.S., Won H.K., Park C.H., Kim G.B., Yu S.D., Choi B.S., Park J.D. (2016). Environmental Exposure to arsenic, lead, and cadmium in people living near Janghang copper smelter in Korea. J. Korean Med. Sci..

[9] Nanda K.P., Kumari C., Dubey M., Firdaus H. (2019). Chronic lead (Pb) exposure results in diminished hemocyte count and increased susceptibility to bacterial infection in *Drosophila melanogaster*. Chemosphere.

[10] Jorissen A., Plum L.M., Rink L., Haase H. (2013). Impact of lead and mercuric ions on the interleukin-2-dependent proliferation and survival of T cells. Arch. Toxicol..

[11] Chibowska K., Baranowska-Bosiacka I., Falkowska A., Gutowska I., Goschorska M., Chlubek D. (2016). Effect of lead (Pb) on inflammatory processes in the brain. Int. J. Mol. Sci..

[12] Baos R., Jovani R., Forero M.G., Tella J.L., Gómez G., Jiménez B., González M.J., Hiraldo F. (2006). Relationships between T-cell-mediated immune response and Pb, Zn, Cu, Cd, and as concentrations in blood of nestling white storks (*Ciconia Ciconia*) and black kites (*Milvus migrans*) from Doñana (southwestern Spain) after the Aznalcóllar toxic spill. Environ. Toxicol. Chem..

[13] Kim M.G., Ryoo J.H., Chang S.J., Kim C.B., Park J.K., Koh S.B., Ahn Y.S. (2015). Blood lead levels and cause-specific mortality of inorganic lead-exposed workers in South Korea. PLoS ONE.

[14] Steenland K., Barry V., Anttila A., Sallmen M., Mueller W., Ritchie P., McElvenny D.M., Straif K. (2019). Cancer incidence among workers with blood lead measurements in two countries. Occup. Environ. Med..

[15] Chaplin D.D. (2010). Overview of the immune response. J. Allergy Clin. Immunol..

[16] Zhao Z.Y., Li R., Sun L., Li Z.Y., Yang R.L. (2004). Effect of lead exposure on the immune function of lymphocytes and erythrocytes in preschool children. J. Zhejiang Univ.-Sci..

[17] Sata F., Araki S., Tanigawa T., Morita Y., Sakurai S., Katsuno N. (1997). Changes in natural killer cell subpopulations in lead workers. Int. Arch. Occup. Environ. Health.

[18] Mishra K.P., Singh V.K., Rani R., Yadav V.S., Chandran V., Srivastava S.P., Seth P.K. (2003). Effect of lead exposure on the immune response of some occupationally exposed individuals. Toxicology.

[19] Massadeh A.M., Al-Safi S. (2005). Analysis of cadmium and lead: Their immunosuppressive effects and distribution in various organs of mice. Biol. Trace Elem. Res..

[20] Fang L., Zhao F., Shen X., Ouyang W., Liu X., Xu Y., Yu T., Jin B., Chen J., Luo W. (2012). Pb exposure attenuates hypersensitivity in vivo by increasing regulatory T cells. Toxicol. Appl. Pharmacol..

[21] McCabe M.J., Lawrence D.A. (1991). Lead, a major environmental pollutant, is immunomodulatory by its differential effects on CD4+ T cells subsets. Toxicol. Appl. Pharmacol..

[22] Yimthiang S., Waeyang D., Kuraeiad S. (2019). Screening for elevated blood lead levels and related risk factors among Thai children residing in a fishing community. Toxics.

[23] Thanapop C., Geater A.F., Robson M.G., Phakthongsuk P., Viroonudomphol D. (2007). Exposure to lead of boatyard workers in southern Thailand. J. Occup. Health Psychol..

[24] https://www.osha.gov/OshDoc/Directive_pdf/CPL_03-00-0009.pdf.

[25] Trzcinka-Ochocka M., Brodzka R., Janasik B. (2016). Useful and fast method for blood lead and cadmium determination using ICP-MS and GF-AAS; validation parameters. J. Clin. Lab. Anal..

[26] Böyum A. (1968). Isolation of mononuclear cells and granulocytes from human blood. Isolation of monuclear cells by one centrifugation, and of granulocytes by combining centrifugation and sedimentation at 1 g. Scand. J. Clin. Lab. Investig. Suppl..

[27] Shi Z., Zhen S., Orsini N., Zhou Y., Zhou Y., Liu J., Taylor A.W. (2017). Association between dietary lead intake and 10-year mortality among Chinese adults. Environ. Sci. Pollut. Res..

[28] Wang X., Ding N., Tucker K.L., Weisskopf M.G., Sparrow D., Hu H., Park S.K. (2017). A Western diet pattern is associated with higher concentrations of blood and bone lead among middle-aged and elderly men. J. Nutr..

[29] Koller L.D. (1979). Effects of environmental contaminants on the immune system. Adv. Vet. Sci. Comp. Med..

[30] Cobbina S.J., Xu H., Zhao T., Mao G., Zhou Z., Wu X., Liu H., Zou Y., Wu X., Yang L. (2015). A multivariate assessment of innate immune-related gene expressions due to exposure to low concentration individual and mixtures of four kinds of heavy metals on zebrafish (*Danio rerio*) embryos. Fish Shellfish Immunol..

[31] Mishra K.P. (2009). Lead exposure and its impact on immune system: A review. Toxicol. In Vitro.

[32] Lawrence D.A. (1981). In vivo and in vitro effects of lead on humoral and cell-mediated immunity. Infect. Immun..

[33] Queiroz M.L., Costa F.F., Bincoletto C., Perlingeiro R.C., Dantas D.C., Cardoso M.P., Almeida M. (1994). Engulfment and killing capabilities of neutrophils and phagocytic splenic function in persons occupationally exposed to lead. Int. Immunopharmacol..

[34] Sen G.C. (2001). Viruses and interferons. Annu. Rev. Microbiol..

[35] Fenga C., Gangemi S., Di Salvatore V., Falzone L., Libra M. (2017). Immunological effects of occupational exposure to lead (Review). Mol. Med. Rep..

[36] Laschi-Loquerie A., Descotes J., Tachon P., Evreux J.C. (1984). Influence of lead acetate on hypersensitivity. Experimental study. J. Immunopharmacol..

[37] Hemdan N.Y.A., Emmrich F., Adham K., Wichmann G., Lehmann I., El-Massry A., Ghoneim H., Lehmann J., Sack U. (2005). Dose-dependent modulation of the in vitro cytokine production of human immune competent cells by lead salts. Toxicol. Sci..

[38] Yang W.-C., Hwang Y.-S., Chen Y.-Y., Liu C.-L., Shen C.-N., Hong W.-H., Lo S.-M., Shen C.-R. (2017). Interleukin-4 supports the suppressive immune responses elicited by regulatory T Cells. Front. Immunol..

[39] Radbin R., Vahedi F., Chamani J. (2014). The influence of drinking-water pollution with heavy metal on the expression of IL-4 and IFN-γ in mice by real-time polymerase chain reaction. Cytotechnology.

[40] Kasten-Jolly J., Lawrence D.A. (2014). Lead modulation of macrophages causes multiorgan detrimental health effects. J. Biochem. Mol. Toxicol..

[41] Hernández-Castro B., Doníz-Padilla L.M., Salgado-Bustamante M., Rocha D., Ortiz-Pérez M.D., Jiménez-Capdeville M.E., Portales-Pérez D.P., Quintanar-Stephano A., González-Amaro R. (2009). Effect of arsenic on regulatory T cells. J. Clin. Immunol..

[42] Gera R., Singh V., Mitra S., Sharma A.K., Singh A., Dasgupta A., Singh D., Kumar M., Jagdale P., Patnaik S. (2017). Arsenic exposure impels CD4 commitment in thymus and suppress T cell cytokine secretion by increasing regulatory T cells. Sci. Rep..

[43] Burchill M.A., Yang J., Vogtenhuber C., Blazar B.R., Farrar M.A. (2007). IL-2 receptor beta-dependent STAT5 activation is required for the development of Foxp3+ regulatory T cells. J. Immunol..

[44] Andrew A.S., Jewell D.A., Mason R.A., Whitfield M.L., Moore J.H., Karagas M.R. (2008). Drinking-water arsenic exposure modulates gene expression in human lymphocytes from a U.S. population. Environ. Health Perspect..

[45] Chen W., Jin W., Hardegen N., Lei K.-J., Li L., Marinos N., McGrady G., Wahl S.M. (2003). Conversion of peripheral CD4+CD25− naive T cells to CD4+CD25+ regulatory T cells by TGF-beta induction of transcription factor Foxp3. J. Exp. Med..

[46] Abu-Eid R., Samara R.N., Ozbun L., Abdalla M.Y., Berzofsky J.A., Friedman K.M., Mkrtichyan M., Khleif S.N. (2014). Selective inhibition of regulatory T cells by targeting the PI3K-Akt pathway. Cancer Immunol. Res..

[47] Li S., Zhengyan Z., Rong L., Hanyun C. (2005). Decrease of CD4+T-lymphocytes in children exposed to environmental lead. Biol. Trace Elem. Res..

